# 临床Ⅰa期肺腺癌N分期上调的危险因素

**DOI:** 10.3779/j.issn.1009-3419.2018.06.07

**Published:** 2018-06-20

**Authors:** 毅 秦, 桐 邱, 云鹏 玄, 艳东 赵, 文捷 矫

**Affiliations:** 266001 青岛，青岛大学附属医院胸外科 Department of Thoracic Surgery, the Affiliated Hospital, Qingdao University, Qingdao 266001, China

**Keywords:** 肺肿瘤, 临床早期, N分期, 影像学, 病理学, Lung neoplasms, Clinical early stage, N upstaging, Radiology, Pathology

## Abstract

**背景与目的:**

即使是临床Ⅰa(cT1N0M0)期肺腺癌的患者, 部分预后也相对较差, 这可能与术后病理学淋巴结转移而导致的N升期有关。本研究旨在分析影响临床早期肺腺癌N分期上调的影像学及病理学因素。

**方法:**

回顾性研究2012年5月-2016年12月于我院行手术治疗的297例临床Ⅰa期肺腺癌的患者, 分析N分期上调组及非上调组的临床一般资料、影像学及病理学资料的差异。

**结果:**

297例cN0肺腺癌中, 250例(84.2%)被证实为pN0, 47例(15.8%)被证实为pN1和pN2。其中, 女性、吸烟史、脉管癌栓、微乳头和实体为主病理亚型的肺腺癌以及影像学中纯实性结节等, 均是N分期上调的影响因素(*P* < 0.05)。*Logistic*回归分析表明:影像学中实性肿瘤、微乳头及实体为主型腺癌和脉管癌栓是临床Ⅰa期腺癌的出现N分期上调的独立危险因素。

**结论:**

病理学脉管癌栓、微乳头和实体为主的肺腺癌以及影像学中纯实性结节是Ⅰa期肺腺癌N分期上调的独立危险因素。临床Ⅰa期的肺腺癌其影像学表现为纯实性结节时, 应进行系统性淋巴结清扫。

## Abstract (en)

**Background and objective:**

In clinical Ⅰa (cT1N0M0) patients, some may have poor prognosis, for it might occur pathologic N1 (pN1) or N2 (pN2) postoperatively.The aim of this study is to determine the radiologicaland pathological factors related to clinical Ⅰa adenocarcinoma.

**Methods:**

The retrospective study was conducted on 297 clinical Ⅰa adenocarcinoma patients resected at our hospital between May 2012 to December 2016.The clinical profiles, radiological and pathological features were analyzed between nodal upstaging group and non-upstaging group.

**Results:**

Of 297 patients treated for cN0 tumors, 250 cases (84.2%) were confirmed postoperatively as having pN0 tumors, and 47 (15.8%) were confirmed as having pN1 or pN2 tumors.Female, low smoking index, micropapillary predominant and solid predominant adenocarcinoma, puresolid tumor and large tumor size were all more frequently seen in the nodal upstaging group than in the pN0 group (*P* < 0.05).*Logistic* regression indicate that radiological solid tumor, micropapillary predominant and solid predominant adenocarcinoma and vessel invasionare the risk factors of nodal upstaging in clinical Ⅰa adenocarcinoma.

**Conclusion:**

Radiological solid tumors, micropapillary predominant and solid predominant adenocarcinoma andvessel invasion are risk factors for nodal upstaging for early stage lung cancer.Radiological solid tumors should perform SLND in Ⅰa adenocarcinomas.

肺腺癌是世界上发病率和死亡率都很高的癌症, 这主要是由于其具有极强的转移侵袭能力。这使得临床Ⅰa期患者, 即使术前影像学评价为淋巴结阴性(cN0), 术后病理学结果也有存在纵膈淋巴结转移(pN2)或肺内淋巴结转移(pN1)转移的可能^[1, 2]^, 这种情况被定义为N分期上调(nodal upstaging)。早期非小细胞肺癌(non-small cell lung cancer, NSCLC)的N分期上调并不少见, 根据Cancer and Leukemia Group B(CALGB 9761)的临床研究^[3]^表明, 临床Ⅰ期NSCLC患者28%出现了术后N分期上调, 这部分患者预后也相对较差。因此, 本研究通过收集2012年5月-2016年12月在青岛大学附属医院住院, 并且行手术切除肺部病损的297例临床Ⅰa期肺腺癌患者, 对患者的性别、年龄、吸烟情况、手术情况、影像学征象以及病理学征象加以数据分析, 旨在了解临床Ⅰa期肺腺癌发生N分期上调的独立危险因素。

## 1 资料与方法

### 1.1 一般资料

来自青岛大学附属医院2012年5月-2016年12月在我院接受手术治疗的297例肺腺癌患者。此研究入组标准如下:①通过完善的术前检查, 考虑为临床Ⅰa期(即cT1N0M0)NSCLC; ②根据国际肺癌研究协会(The International Association for the Study of Lung Cancer, IASLC)/美国胸科学会(American Thoracic Society, ATS)/欧洲呼吸学会(European Respiratory Society, ERS)的肺腺癌分类标准, 病理学诊断为肺腺癌; ③肺部肿瘤为单发; ④进行了标准的肺叶切除术或亚肺叶切除术+系统性淋巴结清扫术(至少清扫10个以上淋巴结); ⑤不纳入行新辅助化疗的患者; ⑥入组患者年龄区间为18岁-70岁。收集的资料包括:①一般资料:性别、年龄、吸烟情况、合并症等; ②手术资料:手术方式、手术切除范围、手术时间、术中出血量等; ③影像学资料:分叶征、毛刺征、强化征、胸膜牵拉征、影像学实性成分等; ④病理学资料:胸膜浸润、脉管癌栓、肺腺癌分型、肿瘤直径等。肺腺癌亚型根据IASLC/ATS/ERS的肺腺癌分类标准分为以下八类:实体成分为主型(Sol)、微乳头成分为主型(micropapillary predominant adenocarcinoma, MIP)、浸润性腺癌变异为主型(VIA)、乳头状为主型(papillary predominant adenocarcinoma, Pap)、腺泡为主型(acinar predominant adenocarcinoma, Aci)、贴壁生长为主型(nonmucinous lepidic predominant adenocarcinoma, Lep)、微浸润性腺癌(minimally invasive adenocarcinoma, MIA)以及原位腺癌(adenocarcinoma *in situ*, AIS)。由于实性成分为主型腺癌以及微乳头成份为主的腺癌其侵袭性较高且预后较差, 因此在进行腺癌亚型分析时, 将这两种亚型分为一组与其他亚型相比较^[4]^。

### 1.2 统计学方法

以N升期作为分组条件, 使用SPSS 22.0进行数据分析, 组间差异比较应用*t*检验、χ^2^检验等分析方法。使用*Kaplan-Meier*方法研究组件总生存时间(overall survival, OS)的差异。应用*Logistic*回归分析N升期上调的影响因素, *P* < 0.05为差异有统计学意义。

## 2 结果

### 2.1 N分期上调是临床Ⅰa期腺癌预后的危险因素

297例患者中, 47例(15.8%)患者出现N分期上调, 250例(84.2%)患者未出现N分期上调, 总体N分期上调率为15.8%。未上调组内平均随访时间为26.3个月, 上调组内平均随访时间为25.2个月。使用*Kaplan-Meier*方法分析N分期上调组与非上调组的总体生存时间, 组间生存时间具有差异(*P* < 0.05)(图 1)。在11例(3.70%)死亡病例中, 所有患者均为癌症相关死亡, 10例为N分期上调组患者, 1例是未发生N分期上调组的患者。

**1:**
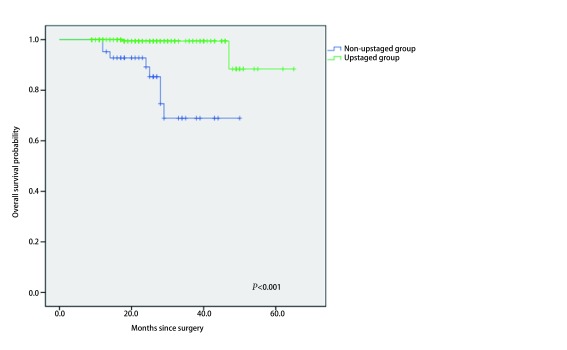
不同N分期总体生存时间 Overall survival curves of patients according to N status.In nodal upstaged patients, 3-year OS of 80.85% (mean OS of 42 months; 95%CI:37-47), while in non-upstaged group, 3-year OS of 99.60% (mean OS of 62 months; 95%CI:60-65) (*P* < 0.000, 1).OS:overall survival.

### 2.2 组间临床资料比较

两组患者性别差异有统计学意义(*P*=0.031), 女性N升期率高于男性(19.00% *vs* 9.28%)。此外, 有吸烟史的患者(一生中连续或累积吸烟6个月或以上者)与无吸烟史的患者相比, N分期上调具有统计学差异。但是, 两组之间手术方式选择没有差异(*P*=0.217), 在非上调组中, 电视胸腔镜辅助胸外科手术、机器人辅助胸外科手术和开放手术的数量分别为173、75和2;在上调组中, 其频数为29、16和2。手术切除范围的差异无统计学意义(*P*=0.190)。两组FEV_1_预测值分别为(2.91±0.67)和(2.78±0.44), 无统计学差异(*P*=0.099)。两组之间肿瘤所在肺叶位置的差异也无统计学意义(*P*=0.171)。本研究中两组患者的高血压病史、结核病史、糖尿病史、类风湿性关节炎病史等合并症的差异均无统计学意义(*P* > 0.05)(表 1)。

**1:** 临床概况 Clinical profiles

	Non-upstaged (*n*=250)	Upstaged (*n*=47)	*P*
Age (yr)	60.22±8.190	59.47±7.544	0.561
FEV_1_, predicted	2.91±0.67	2.78±0.44	0.099
Smoking history			0.007
Yes	68	5	
No	182	42	
Gender			0.031
Male	88	9	
Female	162	38	
Type of resection			0.217
Sublobectomy	32	2	
Lobectomy	220	49	
Surgical approaches			0.190
VATS	173	29	
RATS	75	16	
Thoractomy	2	2	
Tumor location			0.171
Left upper lobe	86	13	
Left lower lobe	36	9	
Right upper lobe	57	11	
Right middle lobe	22	2	
Right lower lobe	49	12	
History of hypertension			0.436
Yes	83	19	
No	167	28	
History of tuberculosisn			0.356
Yes	11	1	
No	239	46	
History of diabetes			0.179
Yes	43	3	
No	207	44	
History of rheumatoid arthritisn			0.543
Yes	2	0	
No	248	47	
VATS:video-assisted thoracic surgery; RATS:robot assisted thoracic surgery.			

### 2.3 组间影像学及病理学资料比较

47例上调的病例, 19例病例出现了强化征, 22例患者出现了分叶征, 10例病例是影像学纯实性结节, 18例病例具有毛刺征。卡方检验结果:影像学实体肿瘤及强化征存在组间差别义(*P* < 0.05), 其中影像学实性成份的统计学意义更为突出(*P*=0.002)。而在病理学中, 两组间肿瘤最大直径分别为(1.85±0.841)cm和(2.46±0.750)cm, 差异有统计学意义(*P* < 0.000, 1), N分期上调组的肿瘤直径较大。微乳头及实性成份为主的患者有15例出现了N分期上调。出现胸膜浸润征象的病例中有27例出现了N分期上调。有脉管癌栓征象的病例, 35例出现了N分期上调。两组病例之间, 胸膜浸润、脉管癌栓、微乳头及实性成份为主的腺癌以及肿瘤直径均有统计学差异(*P* < 0.05)(表 2)。

**2:** 放射学和病理学特征的比较 Comparison on radiological and pathological characteristics

	Non-upstaged (*n*=250)	Upstaged (*n*=47)	*P*
Enhanced sign			0.044
Yes	65	19	
No	185	28	
Lobulation sign			0.083
Yes	84	22	
No	166	25	
Tumor pattern			0.002
Solid tumor	35	10	
Mixed tumor or pure GGO	115	37	
Spicular sign			0.989
Yes	96	18	
No	154	29	
Subtype of adenocarcinoma			< 0.000, 1
MIP or Sol predominant adenocarcinoma	42	15	
Other subtypes of adenocarcinoma	208	32	
Visceral pleural involvement			0.002
Yes	84	27	
No	166	20	
Vessel invasion			< 0.000, 1
Yes	12	35	
No	238	12	
Tumor size (cm)	1.85±0.841	2.46±0.750	< 0.000, 1
Other subtypes of adenocarcinoma include:AIS:adenocarcinoma *in situ*; MIA:minimally invasive adenocarcinoma; Lep:nonmucinous lepidic predominant adenocarcinoma; Aci:acinar predominant adenocarcinoma; Pap:papillary predominant adenocarcinoma; MIP:micropapillary predominant adenocarcinoma; Sol:solid predominant adenocarcinoma.			

### 2.4 多因素方差分析

将性别、吸烟史、强化征、胸膜浸润、脉管癌栓、微乳头及实性成份为主的腺癌以及肿瘤直径等影响因素放入多因素方差分析, 结果示:影像学纯实性结节, 腺癌亚型和脉管癌栓是N分期上调的独立危险因素(*P* < 0.05), 而性别、吸烟史、强化征、胸膜浸润不是N分期上调的独立危险因素(*P* > 0.05)。术前影像学评价为纯实性结节以及病理学存在脉管癌栓的病人容易发生N分期上调。此外, 与其他亚型的腺癌相比, 微乳头及实性成份为主的腺癌更容易发生N分期上调(表 3)。

**3:** N分期上调相关的多因素方差分析 Multivariate analysis for factors associated with nodal upstaging

Covariates	*P*	Hazard ratio	95%CI
Tumor pattern	0.028	2.597	1.109-6.061
Subtype of adenocarcinoma	< 0.000, 1	2.071	1.466-2.925
Vessel invasion	< 0.000, 1	10.010	1.907-12.015
Enhanced sign	0.793	1.147	0.412-3.191
Visceral pleural involvement	0.188	2.012	0.198-1.374
Tumor size	0.669	1.154	0.598-2.226
Smoking history	0.089	0.028	0.043-1.244
Gender	0.125	0.338	0.084-1.352

## 3 讨论

TNM分期是NSCLC预后的最准确的预测指标, 而N分期上调直接将术前预估的Ⅰ期NSCLC上升至Ⅱ期甚至Ⅲ期, 对于患者的治疗产生了极大的影响, 根据NCCN指南, 术后pN1和pN2的患者需要接受辅助化疗, 即使是早期NSCLC, 也有可能同时进行放疗。本研究中, 证实了临床Ⅰa肺腺癌淋巴结转移患者的预后较差(图 1), 因此, 通过研究临床早期肺腺癌淋巴结隐匿性转移的临床及病理危险因素, 对于加深对NSCLC的诊治具有极为重要的意义。为了研究淋巴结转移的危险因素, 我们进行了统计分析并得出结论:影像学实体瘤, 微乳头和实体成分为主的腺癌以及脉管癌栓是发生N升期的独立危险因素。最近有学者调查了2, 268例患者, 分析了临床病理特征与N分期上调的关系。通过卡方检验发现肿瘤大小, 吸烟状况, 脏层胸膜浸润, 腺癌亚型和性别与淋巴结累及有关^[5]^。在我们的研究中, 得出与之相同的结论:肿瘤直径大小、吸烟状况、VPI状态、腺癌亚型和性别以及脏层胸膜浸润, 被发现与N分期上调有关, 同时影像学实性肿瘤和脉管癌栓均与N分期上调相关(表 1和表 2)。当进一步行多变量分析时, 只有影像学中实体肿瘤, 腺癌亚型和脉管癌栓是淋巴结转移的独立危险因素(表 3)。

Rocha等^[6]^研究了109例临床Ⅰ期/Ⅱ期肺癌患者, 并得出结论:位于下叶的早期NSCLC与N分期上调有关。其研究价值有限, 因为文章并未对影响N升期的因素加以分析。而另一项研究认为:患有结核病史、类风湿性关节炎病史、糖尿病史的患者较易出现淋巴结分期不准确(上调或下调)。但是, 出现这种现象的原因是疾病本身导致淋巴结粘连较重, 术者无法进行准确的术式, 而并非淋巴结侵袭本身的特征。本文进行了并发症研究, 结果并未出现差异(表 1)^[2, 7-9]^。Lee等^[10]^对于N升期进行了详尽的研究, 实验结果示:肿瘤直径大于2 cm的中心型NSCLC且SUVmax≥4, 这种肿瘤较易发生N2组淋巴结转移。该实验也指出, 腺癌是淋巴结发生N升期的危险因素, 但却未进行详尽研究。我们的实验根据最新版腺癌分型, 对腺癌进行了分组, 将侵袭性较强的Sol以及MIP与其他类型腺癌分开比较, 从而得出结论Sol和MIP组较易发生N分期上调。

目前尚未有文献指出影像学纯实性结节、脉管癌栓与肺腺癌N分期上调存在联系。但许多研究影像学实性结节的文献指出, 影像学中纯实体肿瘤较纯毛玻璃影及混合密度结节表现出更强的侵袭性^[11-13]^。我们的研究认为, 影像学纯实性结节较容易发生N分期上调, 这可能与其侵袭性密切相关。同样的道理, 目前有文章指出, 即使是病理Ⅰ期的NSCLC患者, 脉管癌栓也是患者预后较差的征象^[14, 15]^。由此可见脉管癌栓是肿瘤侵袭性较强的指标, 由于这个特性, 有学者提出将脉管癌栓纳入T升期的范畴, 但仍存在争议。根据我们文章的结论, 认为脉管癌栓是N升期的独立危险因素, 这足以证实其侵袭性强的特性。

根据我们的实验结果, MIP和Sol为主的腺癌、影像学纯实性结节以及脉管癌栓是临床Ⅰa期肺腺癌N分期上调的危险因素, 我们将这一结论与临床相结合, 指导术中淋巴结的处理。目前认为关于临床Ⅰa期NSCLC的手术治疗, 系统性淋巴结清扫与淋巴结采样的效果文献报道不一, 有作者认为系统性淋巴结清扫效果优于淋巴结采样^[16, 17]^, 亦有作者认为两者之间效果相仿, 因为在生存率上无显著差异^[18]^。但一个共同点均认为与淋巴结采样效果明显优于单纯肿瘤手术切除, 对减少术后复发、提高患者的生存率有益^[18, 19]^。根据本实验结论, 临床中在对术前N0考虑肺癌的患者进行手术时, 可以进行如下评估:影像学评估为小于3 cm纯实性结节, 且术中做快速病理检查结果为腺癌, 则应行系统淋巴结清扫术, 而非淋巴结采样或区域性淋巴结清扫, 因为其存在淋巴结隐匿性转移的风险。如果腺癌的亚型可以在术中确诊, Sol和MIP为主要成分的腺癌也应该进行系统淋巴结清扫。

综合本实验研究的结果, 我们可以得出结论:病理学脉管癌栓、微乳头和实体为主的肺腺癌以及影像学中纯实性结节是Ⅰa期肺腺癌N分期上调的独立危险因素。临床Ⅰa期的肺腺癌其影像学表现为纯实性结节时, 应进行系统性淋巴结清扫。
